# Biological functions and potential implications of circular RNAs

**DOI:** 10.7555/JBR.36.20220095

**Published:** 2022-10-28

**Authors:** Lan Ma, Haiyan Chu, Meilin Wang, Zhengdong Zhang

**Affiliations:** 1 Department of Genetic Toxicology, The Key Laboratory of Modern Toxicology of Ministry of Education, Center for Global Health, School of Public Health, Nanjing Medical University, Nanjing, Jiangsu 211166, China; 2 Department of Environmental Genomics, Jiangsu Key Laboratory of Cancer Biomarkers, Prevention and Treatment, Collaborative Innovation Center for Cancer Personalized Medicine, Nanjing Medical University, Nanjing, Jiangsu 211166, China

**Keywords:** circular RNAs, biogenesis, mechanisms, database, biomarkers

## Abstract

Circular RNAs (circRNAs) are characterized by a covalent closed-loop structure with an absence of both 5′ cap structure and 3′ polyadenylated tail. Numerous studies have found that circRNAs play an important role in various diseases and have a variety of biological regulatory mechanisms, including acting as microRNA sponges, interacting with proteins, modulating the expression of related genes and translating into peptides or proteins. CircRNAs have also been used as biomarkers for a number of diseases, which could improve clinical practice. This review summarizes the most recent advances in biogenesis and knowledge of the biological functions of circRNAs as well as the related bioinformatics databases. We specifically describe developments in understanding of circRNA functions in the field of environmental exposure-induced diseases. Finally, we focus on potential clinical implications of circRNAs to facilitate their clinical transformation into disease treatment.

## Introduction

Circular RNAs (circRNAs), members of the noncoding RNA family, are a class of endogenously expressed regulatory RNA molecules characterized by a covalently-closed loop structure without 5′ cap structure and 3′ polyadenylated tail. Viroids are a type of circRNA molecules first discovered in 1976^[1]^. For many decades thereafter, viroids were only thought to be relatively scarce, resulting from alternative splicing errors that can occur during the transcription phase. Later in 1993, testis-specific circRNA, derived from the sex-determining region Y (*Sry*) gene in mouse testis, was thought to have a possible function^[2]^.

Advances in high-throughput RNA sequencing technologies and bioinformatics algorithms have enabled researchers to describe circRNAs in-depth for their identification and potential functions^[3]^. We now know that circRNAs are involved in cell proliferation, differentiation, apoptosis and invasion and therefore throughout disease progression^[4–7]^. Especially, a large number of circRNAs have been linked to cancer progression^[8]^. More importantly, circRNAs have also been found to influence tumor cell survival after exposure to chemotherapy drugs. Thus, circRNAs may become a novel target in cancer therapeutics^[9–10]^. Therefore, further exploring the molecular mechanisms of circRNAs could open up a new area in the targeted therapy and drug resistance research.

This review summarizes recent biogenesis research and biological functions involved in circRANs as well as explores the related bioinformatics databases to add to the existing circRNA knowledge base. We also outline potential implications of this research for diagnostics and therapeutics.

## Biogenesis mechanisms and the characteristics of circRNAs

CircRNAs are mainly formed from their precursor messenger RNAs (mRNAs) through a unique reverse splicing process. Based on differences in the composition and cycling mechanisms, circRNAs are divided into three main types, *i.e.*, exonic circRNAs (EcircRNAs), exon-intron circRNAs (EIciRNAs), and circular intronic RNAs (ciRNAs)^[11–13]^. Additionally, the fused circRNAs (f-circRNAs), which are generated by way of chromosomal rearrangements^[14]^, and the read-through circRNAs (rt-circRNAs), which are produced by looping two adjacent gene exons on the same strand^[15]^, have been recently identified.

CircRNA biogenesis is regulated by a variety of factors, such as enzymes, intronic sequences, and RNA-binding proteins (RBPs). Intronic complementary sequences, which contain splicing sites along with short-inverted repeats (*e.g.*, *Alu* elements), and complementary base-pairing flanking the back-splicing sites, are essential for circularization^[16–18]^. In addition, alternative back-splicing of inversely repeated *Alu* elements in flanking introns and competition between RNA pairings can lead to an alternative circularization, resulting in the formation of multiple circRNAs transcripts from a single gene^[12,19]^.

RBPs play important roles in circRNA generation by promoting or suppressing intron pairing. As a major regulator of circRNA biogenesis, quaking (QKI) binds upstream and downstream of the circRNA-forming exons in pre-mRNA to stimulate circRNA circulation during the epithelial-mesenchymal transition (EMT) process^[20]^. The fused in Sarcoma (FUS) depletion and mutation affect circRNA biogenesis in mouse embryonic stem cell-derived motor neurons by binding to specific sequence sites of flanking introns and linking them together^[21]^. Splicing factor proline/glutamine rich (SFPQ) is an essential regulator for Distal-Alu-Long-Intron (DALI) circRNA production in mammals by controlling and enforcing accurate splicing of long introns^[22]^. Conversely, adenosine deaminase acting on RNA 1 (ADAR1) can bind to double-stranded RNA and disrupt RNA pairing by performing A-to-I editing of inverted-repeat *Alu* elements that flank circRNA-forming exons, thereby inhibiting circRNA biogenesis^[23–24]^. The DExH-box helicase 9 (DHX9) protein has been shown to reduce the production of *Alu*-dependent circRNAs *via* affecting intronic base pairing^[25]^.

Transcriptional factors (TFs) have also been found to regulate circRNA expression by modifying the transcription of the host gene^[26]^. Through bioinformatics prediction of TRCirc^[27]^ and experimental verification, circSPARC expression is controlled by the TF CTCF, which in turn regulates gene expression in human diseases^[28]^. Another study has demonstrated that the TF, TFAP2C, induces circIL4R upregulation by transcriptional regulation of its host gene^[29]^. Therefore, the specific mechanisms involved in circRNA biogenesis have not been fully described. Further studies are needed to generate a better understanding of circRNA biogenesis.

## Functional classification and potential implications of circRNAs

**Figure 1:**
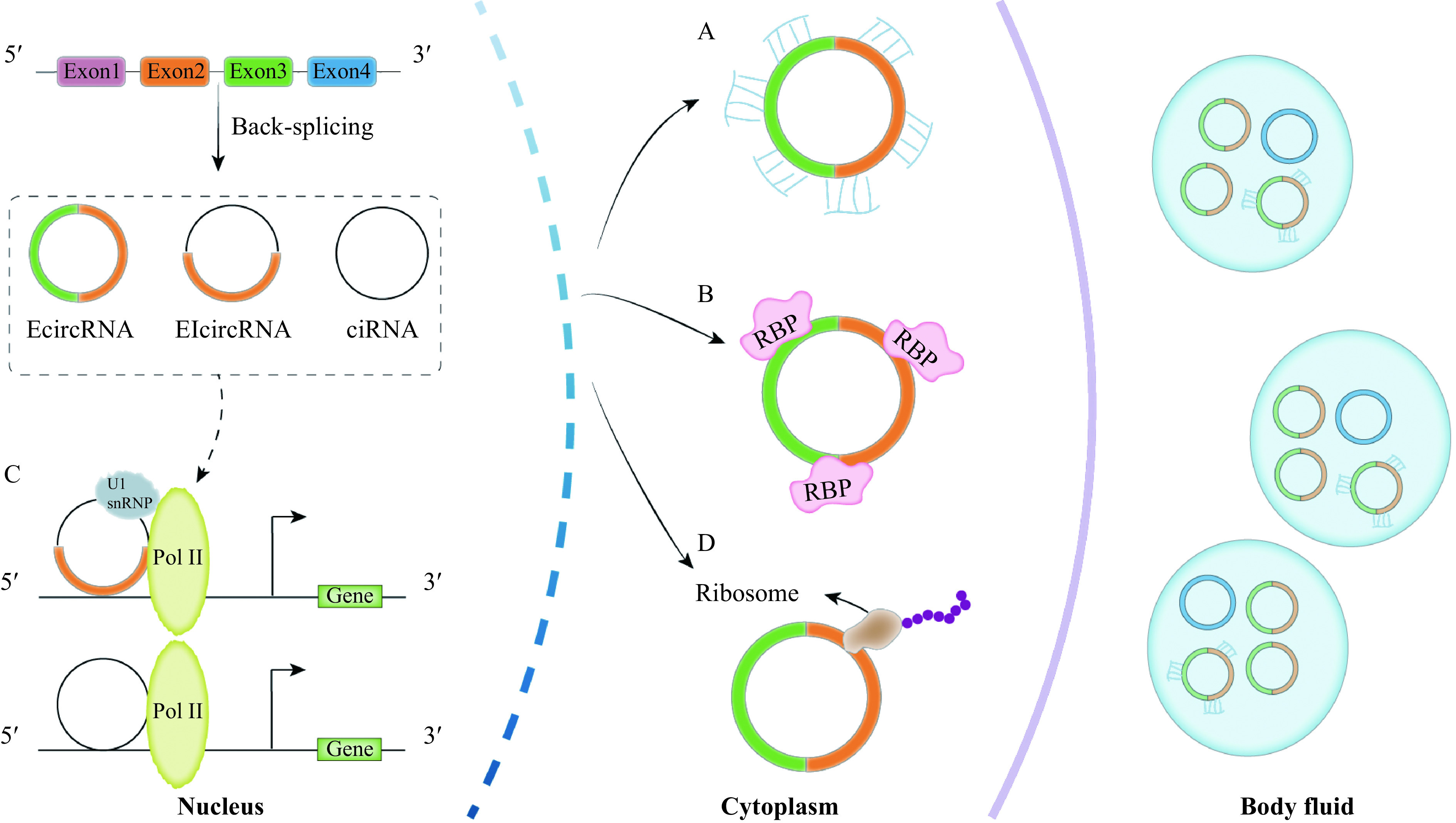
Potential functions and characteristics of circRNAs.

Recently studies have shown the functional importance of circRNAs in various biological and pathological processes^[30]^. Several other cellular processes also appear to be regulated by circRNAs, including chromatin remodeling, transcriptional regulation, translational regulation, RNA stability, and scaffolding. Here, we discuss only a few examples of the functions involved in classification, and we provide some background of the most recent developments in our understanding of circRNA functions and in the field of environmental exposure-induced diseases (***Fig. 1***).

### CircRNAs act as microRNA sponges

MicroRNA** (**miRNA) sponging effect is the most extensively studied function of circRNAs^[31]^. CircRNAs are rich in miRNA binding sites and can bind to complementary sequences within miRNAs, serving as competitive endogenous RNA that regulates gene expression. For instance, CDR1as, also known as ciRS-7, is highly expressed in the mammalian brain and contains more than 70 conserved seed matches for miR-7^[32–33]^. CDR1as transcripts are biologically active by interaction with miR-7 *in vitro* and *in vivo*. Interestingly, CDR1as has one nearly perfectly complementary binding site for miR-671. Therefore, miR-671 can mediate CDR1as slicing, suggesting that CDR1as may release miR-7 to inhibit this transcript. Additionally, deleting CDR1as expression in mice leads to miR-7 down-regulation and up-regulation of miR-671. Similarly, CDR1as can modulate the EMT process and appears to participate in silica-induced pulmonary fibrosis by sponging miR-7^[34]^.

Many other circRNAs can also act as miRNA sponges in environmental exposure-related diseases. For example, circTXNRD1 promotes particulate matter-induced inflammation in human bronchial epithelial cells by regulating the circTXNRD1-miR-892a-COX-2 pathway^[35]^. CircBbs9 binds miR-30e-5p to increase activation of NLRP3 inflammasome, thereby promoting inflammatory responses in mice with PM_2.5_-induced chronic obstructive pulmonary diseases^[36]^. Besides, circ-SHPRH, a circRNA from *SHPRH*, suppresses cadmium-exposure transformation of human bronchial epithelial cells (BEAS-2B) by acting as a sponge of miR-224-5p and regulating QKI expression under cadmium treatment^[37]^. Exosomal circ_100284 derived from the transformed cells exposed to arsenite is involved in the malignant transformation of human hepatic cells, which promotes an accelerated cell cycle and proliferation of normal liver cells *via* miR-217 regulation of EZH2^[38]^. Taken together, these studies suggest that circRNAs modulate disease progression by regulating the expression of miRNA targets.

### CircRNAs can interact with proteins

Some circRNAs have exhibited the capacity to act as protein sponges, scaffolds, and recruiters in multiple pathophysiological processes^[39–40]^. RBPs are a broad class of proteins that bind to RNA molecules, associating with the metabolic processing of RNAs^[41]^. A previous study has shown that circDnmt1 can promote the nuclear translocation of p53 and Auf1 by binding to these proteins in breast cancer cells^[42]^. The accumulation of p53 in the nucleus induces cellular autophagy, whereas nuclear translocation of Auf1 actually increases Dnmt1 expression, causing autophagy and promoting tumor growth. In the cytoplasm, circPABPN1 has also been found to regulate cell proliferation by serving as a decoy for HuR^[43]^. Similarly, circFoxo3 can interact with CDK2 and p21 to inhibit cell cycle progression from G1 to S phase in cancer cell lines^[44]^, or facilitate cardiac senescence by associating with anti-aging proteins ID1 and E2F1 as well as stress-related proteins FAK and HIF1α in the mammalian heart^[45]^. Another study reveals that circNSUN2 modulates the cytoplasmic exportation of a ternary RNA-protein complex (*i.e.*, circNSUN2/IGF2BP2/HMGA2), while enhancing the stability of *HMGA2* mRNA, which in turn promotes colorectal cancer metastases^[46]^.

Considering their functions as protein sponges that regulate biological processes in environmental exposure-related diseases, circ_406961 inhibits the activation of STAT3/JNK pathways *via* sponging ILF2 protein, thereby suppressing the PM_2.5_-induced BEAS-2B cells inflammatory response^[47]^. CircHECTD1 can also interact with ZC3H12A to prevent SiO_2_-induced changes in M1/M2 macrophage phenotypes and can decrease cellular viability, while HECTD1 mediates ZC3H12A expression *via* ubiquitination to promote macrophage polarization^[48]^.

Owing to advances in RNA sequencing technologies and evolving bioinformatics approaches, crosslinking-immunoprecipitation and high-throughput sequencing, RNA pull-down and RNA immunoprecipitation are mainly used to analyze the interactions between circRNAs and proteins, which help us to develop our understanding of circRNAs functions.

### CircRNAs can modulate expression of the related genes

CircRNAs, especially intron-derived circRNAs, can promote gene expression by interacting with chromatin remodeling complexes and increasing RNA polymerase Ⅱ activity^[13,49]^. For instance, ci-ankrd52 has been found to function as a positive regulator of the elongation Pol Ⅱ complex and to play an important role in the efficient transcription of its parent gene^[11]^. CircTulp4 regulates the transcription of its host gene, Tulp4, by directly interacting with U1 small nuclear ribonucleoprotein particle (snRNP) and binding to RNA polymerase Ⅱ. Downregulation of circTulp4 and Tulp4 influences nervous system functions, and therefore participates in the development of Alzheimer's disease^[50]^. In the nucleus, circRNA FECR1 binds to the FLI1 promoter and recruits TET1 demethylase in order to induce DNA demethylation, which in turn regulates gene expression. It is suggested that, FECR1 may regulate breast cancer cell metastasis by coordinating DNA methylation and demethylation in target genes involved in tumor growth^[51]^.

Additionally, circEsyt2 directly binds to the RNA splicing factor PCBP1, and regulates its nuclear translocation, subsequently affecting the alternative splicing of p53 and creating an altered expression of p53 target genes. This influences vascular smooth muscle cells' phenotypic switching as well as vascular remodeling^[52]^. Interestingly, circYap can negatively control the translation of its parent gene by suppressing the formation of translational initiation machinery, and therefore circYap can actually decelerate breast cancer cell progression^[53]^. However, it remains to be seen whether gene transcription can be modulated by circRNA in environmental exposure-related diseases.

### CircRNAs can be translated into peptides or proteins

Dozens of circRNAs have been exported to the cytoplasm as templates for protein synthesis, and functional circRNA-encoded proteins have been found to be involved in various diseases^[54]^. CircRNAs can be translated through cap-independent or cap-dependent mechanisms, including internal ribosome entry site (IRES) initiation mode, N6-methyladenosine (m^6^A) internal ribosome entry site (MIRES) initiation mode, and rolling translation mechanism (RCA)^[55]^.

CircRNAs with IRES elements and AUG sites have the potential to translate functional peptides or proteins^[54]^. Circ-ZNF609, which specifically regulates myoblast proliferation, contains a 753-nt long open reading frame (ORF), according to a number of bioinformatics tools^[56]^, while, the 30 kDa protein produced from circ-ZNF609 in an IRES-like sequence (UTR)-directed mechanism does not contain the zinc-finger domain. This suggests that this protein may have a completely different function, compared to the product of linear RNA. Similarly, Zhang *et al* identified an 87-amino acid polypeptide (PINT87aa), encoded by the second exon of the long non-coding RNA transcript (LINC-PINT)-formed circPINT^[57]^. Functionally, PINT87aa (not its corresponding circRNA) interacts with the polymerase complex-associated factor 1 (PAF1) and controls glioblastoma cell proliferation *in* *vitro* and *in vivo*. In addition, SHPRH-146aa is a novel protein with 146 amino acids generated from circSHPRH, and the majority of its amino acid sequence is the same as the SNF2 domain of SHPRH^[58]^. Surprisingly, the SHPRH-146aa protects the SHPRH protein (190 kDa) from degradation, which infers that SHPRH-146aa may be a protective 'decoy' for SHPRH.

As the most abundant mRNA post-transcriptional modification, m^6^A in the 5′ UTR can function as an alternative to the 5′ cap to stimulate mRNA translation^[59]^. The m^6^A motif "RRACH" (R = G or A; H = A, C or U) enriched in circRNAs is sufficient to drive protein translation in a cap-independent manner by acting on the translation initiation factor eIF4G2 and the m^6^A reader YTHDF3^[60]^. Under the heat shock stress, YTHDF2 in the nucleus maintains 5′ UTR methylation of stress-induced transcripts by competing with the m^6^A "eraser" fat mass and obesity-associated protein^[61]^.

In addition to circRNAs containing IRESs or m^6^A sites, proteins can also be synthesized from circRNAs by way of the RCA mechanism^[62]^. CircRNAs containing longer ORF and initiation codon sequences enable continuous translation termed the RCA mechanism. For example, the rolling-translated EGFR protein translated from circEGFR consistently activates oncogenic EGFR signaling by sustaining EGFR membrane localization in glioblastoma^[63]^.

Till now, several studies have shown that circRNA-encoded proteins exhibit similar or different localizations and physiological functions to host proteins. While the roles of peptides/proteins encoded by circRNAs in environmental exposure-related diseases have not been reported, there are still more regulatory mechanisms underlying translatable circRNAs needs to be addressed in the future.

## Databases for circRNAs and functional predictions

With the improvement of high-throughput RNA sequencing technology, many databases have been developed to identify circRNAs^[64–80]^, such as circBase, CircInteractome, CSCD, CircAtlas, circRNADb, CircNet, and CircR2Disease (***Table 1***).

**Table 1:** Details of databases for circRNAs and functional predictions

Database	Description	Organism	Website
circBase^[64]^	The database contains public circRNA datasets and the comprehensive annotation of circRNA sequences	Six organisms^a^	http://circrna.org/
circBank^[65]^	The database is a comprehensive database of human circRNA which includes more than 140 000 human annotated circRNA from different source	Human	http://www.circbank.cn/
CIRCpedia v2^[66]^	The database contains circRNA annotations from over 180 RNA-seq datasets across six different species	Six organisms^b^	http://yang-laboratory.com/circpedia/
deepBase v3.0^[67]^	The database provides identification, expression, evolution and function of circRNAs from deep-sequencing data	Four organisms^c^	https://rna.sysu.edu.cn/deepbase3/
CircInteractome^[68]^	The database enables the prediction and mapping of binding sites for RBPs and miRNAs on reported circRNAs	Human	http://circinteractome.nia.nih.gov/
CircNet 2.0^[69]^	The database emphasizes on exploring the regulatory network of circRNAs in a wide array of cancers	Human	https://awi.cuhk.edu.cn/~CircNet
CircAtlas^[70]^	The database describes a novel multiple conservation score, co-expression, and regulatory networks for circRNA annotation and prioritization	Six organisms^d^	http://circatlas.biols.ac.cn/
starBase v2.0^[71]^	The database is designed for decoding the interaction networks of lncRNAs, miRNAs, competing endogenous RNAs, RNA-binding proteins and mRNAs from large-scale CLIP-Seq data	Three organisms^e^	https://starbase.sysu.edu.cn/starbase2/
circRNADb^[72]^	The database is a comprehensive database providing the protein-coding annotations for human circRNAs	Human	http://reprod.njmu.edu.cn/cgi-bin/circrnadb/circRNADb.php
TransCirc^[73]^	The database predicts coding potential of circRNAs and the putative translation products	Human	https://www.biosino.org/transcirc/
riboCIRC^[74]^	The database allows researchers to explore, analyze, and visualize translatable circRNAs for multi-species	Six organisms^f^	http://www.ribocirc.com/
CSCD2^[75]^	The database provides comprehensive resources for cancer-specific circRNAs with enhanced functional modules	Human	http://geneyun.net/CSCD2/
CircR2Disease v2.0^[76]^	The database investigates the roles of dysregulated circRNAs in various diseases and further explores the posttranscriptional regulatory function in diseases	Five organisms^g^	http://bioinfo.snnu.edu.cn/CircR2Disease_v2.0
circMine^[77]^	The database develops 13 online analytical functions to comprehensively investigate these datasets to evaluate the clinical and biological significance of circRNAs	Human	http://www.biomedical-web.com/circmine/
circ2Traits^[78]^	The database is a comprehensive database for circular RNA potentially associated with disease and traits	Human	http://gyanxet-beta.com/circdb/
exoRBase 2.0^[79]^	The database provides an attractive platform for the identification of novel exLR signatures from human biofluids	Human	http://www.exoRBase.org
PlantCircNet^[80]^	The database serves as a resource to query detailed information of specific plant circRNAs	Plant	http://bis.zju.edu.cn/plantcircnet/index.php
^a^Six organisms: human, mouse, *C. elegans*, *D. melanogaster*,* L. chalumnae*, and *L. menadoensis*; ^b^Six organisms: human, mouse, rat, zebrafish, fly, and worm; ^c^Four organisms: human, mouse, *C. elegans*, and *D. melanogaster*; ^d^Six organisms: human, macaque, mouse, rat, pig, and chicken; ^e^Three organisms: human, mouse, and *C. elegans*; ^f^Six organisms: human, mouse, rat, *C. elegans*, drosophila, and zebrafish; ^g^Five organisms: human, mouse, rat, chicken, and *C. elegans*. circRNA: circular RNA.			

Based on the content of these circRNA databases, they could be divided into four categories. The first category is comprehensive databases, which include various types of circRNA information in multiple species, such as the circBase, circBank, and CIRCpedia (v2). Currently, the circBase contains the basic information of circRNAs, and can be used to predict miRNA binding sites, target sites of RNA binding proteins, and ribosome profiling tracks^[64]^. CircBank contains more than 140 000 human annotated circRNAs and multiple new circRNA featuress including predicted miRNA binding sites, the potential of circRNA protein coding, circRNA conservation and circRNA methylation^[65]^.

Subsequently, some available databases that integrate different kinds of circRNA-related data to assist functional research of circRNAs are classified as second category sources. CircInteractome facilitates the prediction and mapping of binding sites for RBPs and miRNAs on reported circRNAs^[68]^. Additional functions, such as the collection of genomic and mature sequences of circRNAs as well as the design of specific circRNA divergent primers and circRNA-targeted siRNAs, are included in this database. CircNet 2.0, launched by Lee *et al*, is an updated database for exploring circRNA-mRNA-gene regulatory networks in cancers^[69]^. Furthermore, the circAtlas stores a total of 1 007 087 circRNAs from six vertebrate species and simulates comprehensive interactions of circRNA-mRNA, circRNA-miRNA, and circRNA-RBP^[70]^. Similarly, the remaining three databases, *i.e.*, circRNADb, TransCirc and riboCIRC, focus on the comprehensive protein-coding annotations for circRNAs^[72–74]^.

The third category includes disease-related circRNAs based on the analysis of high-throughput gene expression profiles that can potentially be used as biomarkers for disease diagnosis, such as cancer-specific circRNA database (CSCD), CircR2Disease, circMine, and Circ2Traits. The CSCD records human cancer-specific circRNAs and provides an integrated platform for exploring the function and regulation of circRNAs in cancers, which consists of 1 013 461 cancer-specific circRNAs, 1 533 704 circRNAs from normal samples and 354 422 circRNAs from both cancer and normal samples^[75]^. CircR2Disease (2.0) can serve as a resource for users to systematically investigate the roles of abnormal circRNAs expression in various diseases, and further explore the post-transcriptional regulatory functions in diseases^[76]^. To assess clinical and biological significance of circRNAs under specific physiological and pathological conditions, circMine complies 136 871 circRNAs, 87 diseases and 120 circRNA transcriptome datasets of 1107 samples across 31 human body sites^[77]^.

The last category is for sources that cannot be classified into any of the aforementioned three categories. They have a general focus on a specific direction and collect a certain type of information related to circRNAs, and the exoRBase 2.0, and PlantCircNet, *etc.*, are the representatives. Additionally, our research group recently constructed a user-friendly web interface to visualize each circRNA in fluids^[81]^. This database focuses on the resource of the circRNAs expression in body fluids from pan-cancer dataset and characterizes their clinical applications in liquid biopsy for cancer diagnosis and prognosis, which can significantly contribute to circRNAs in cancer research.

Notably, these databases comprehensively summarize the available information on circRNAs and prioritize functional or disease-related candidate circRNAs, which will also help to decipher their molecular behaviors underlying diseases. There are still some limitations. For instance, the name of the same circRNA in circBase and circBank is different, which may cause confusion. Therefore, a uniform circRNA naming system is required. Meanwhile, given that the current databases are based on many computational approaches to reveal those circRNA-related functional roles or circRNA-disease associations, there is still much room for improvement in prediction accuracy and validation.

## Clinical significance of circRNAs

CircRNAs have received considerable attention in the fields of growth and development, life processes and other diseases^[82–83]^. Evidence has also demonstrated that circRNAs are abundant and stable in exosomes, which can be detected in the circulation and urine^[84]^. Exosomal circRNAs can be shared between cells and have multiple functions, such as promoting inflammatory responses, modulating immunity, regulating cancer cell proliferation, invasion and metastasis as well as in drug resistance^[85–86]^. Several studies have shown that circRNAs might be potential clinical biomarkers for early diagnosis and prognosis as well as promising therapeutic targets^[83,87]^.

### CircRNAs are promising biomarkers for diagnosis and prognosis

As diagnostic markers, dysregulation of circ_014924, circ_006603 and circ_003982 are linked to lung inflammation caused by polystyrene microplastics^[88]^. CircZC3H4 and circHECTD1, which are up-regulated in silicosis patients, may be biomarkers for early diagnosis of silicosis^[89–90]^. Meanwhile, hsa_circ_0058493 serves as a new and promising biomarker for diagnosis of both silicosis and idiopathic pulmonary fibrosis by affecting the EMT process to inhibit the expression of fibrotic molecules^[91]^. Furthermore, an 8-circRNA biomarker panel has been established to serve as potential diagnostic biomarkers for the early detection of gastric cancer^[92]^. As a promising predictor in colorectal cancer diagnosis, exosomal circLPAR1 suppresses colorectal cancer development through decreasing BRD4 *via* METTL3-eIF3h interaction^[93]^.

As a prognostic marker, circIARS in tumor cell-derived exosomes regulates endothelial cell permeability and promotes tumor metastasis^[94]^. Exosomal circIARS expression in pancreatic ductal adenocarcinoma (PDAC) tissues and plasma exosomes is higher than that in the control groups, which suggests that exosomal circRNAs may be an important indicator for early diagnosis and prognostic prediction of PDAC. Down-regulated circRPN2 in highly metastatic hepatocellular carcinoma (HCC) cell lines and HCC tissues is related to shorter overall survival and higher rates of cumulative recurrence, which indicates that circRPN2 might be a novel indicator of HCC prognosis^[95]^. Similarly, another recent study on breast cancer shows that an elevated expression of autophagy-associated circCDYL in the tumor tissues and serum of patients is correlated with higher tumor burden, shorter survival and poorer clinical response to therapy^[96]^. Collectively, the roles of circRNAs in molecular markers could therefore lead to the identification of potential applications as novel biomarkers for disease diagnosis and prognosis.

### CircRNAs can act as therapeutic targets

Due to their specific properties, circRNAs hold potentials for prevention and treatment in biomedical applications. Several approaches have been developed to deliver circRNAs to target organs or tissues, including nanoparticles, exosomes, adeno-associated viruses, and lentiviral vectors^[97–98]^.

As an oncogenic circRNA in HCC, circMDK promotes the progression of HCC *via* the miR-346/874-3p-ATG16L1 axis, resulting in activation of the PI3K/AKT/mTOR pathway, while PAEs-mediated nanoparticles delivery of circMDK siRNA inhibits HCC proliferation and metastasis *in vivo*^[99]^. Interestingly, mitochondria-specific delivery of circSCAR alleviates high-fat diet-induced cirrhosis and insulin resistance in mice and serves as a therapeutic target for nonalcoholic steatohepatitis^[100]^. Engineered circSCMH1-extracellular vesicles can promote functional recovery after stroke in non-human primates and warrant further research and development^[101]^. In addition, exon-derived circRNA-vgll3 functions in the osteogenic differentiation of adipose-derived mesenchymal stem cells (ADSCs) through a circRNA-vgll3/miR-326-5p/Itga5 pathway, and circRNA-vgll3-modified ADSCs can markedly enhance new bone formation *in vivo*, indicating that circRNAs-engineered ADSCs hold potential for repairing non-healing bone defects^[102]^.

Regarding drug resistance in breast cancer, circUBE2D2 may provide a promising therapeutic target^[103]^. CircUBE2D2 is up-regulated in exosomes isolated from tamoxifen-resistant breast cancer cells. Exosomes increase the resistance of breast cancer cells to tamoxifen by mediating the transfer of circUBE2D2 that interacts with miR-200a-3p to avoid drug resistance. Moreover, Qu *et al* established a novel approach using circRNAs to produce SARS-CoV-2-related interventions, including vaccines, therapeutic nanobodies, and hACE2 decoys^[104]^, in which the circRNA vaccines that encoded the trimeric RBD of the spike protein elicited potent neutralizing antibodies and T cell responses, providing effective protection against SARS-CoV-2 in both mice and monkeys. Besides, circRNA^RBD-Delta^ vaccines induced broad-spectrum protection against the current variants of concern SARS-CoV-2. In summary, circRNAs might be applied as an effective and safe platform for vaccination against viral infection, including SARS-CoV-2 emerging variant, which can reduce the potential side effects of vaccine-associated respiratory diseases more effectively^[105]^.

## Conclusions and perspectives

Recent studies have revealed that thousands of circRNAs are aberrantly expressed in tissue and organ development, which may play roles in various disease processes, such as neuro-degeneration and cancer development. In general, the four main regulatory mechanisms and biological functions of circRNAs are microRNA sponges, circRNAs-proteins interactions, altered expression of related genes, and translation template. Due to the complexity of disease pathogenesis, the exact function of these circRNAs remains unclear. Advances in biological research techniques will improve our understanding of the circRNA functions and will provide insight into the molecular mechanisms associated with circRNAs.

Given their stability, conserved, and cell/tissue-specific expression patterns, circRNAs may serve as biomarkers to facilitate the diagnosis of diseases as well as to predict responses to certain treatments. CircRNAs are abundant in body fluids, including blood, saliva, urine, and exosomes, which makes them desirable noninvasive biopsy biomarker candidates. For instance, the expression profiles of exosomal circRNAs in patients differ from healthy groups, indicating that exosomal circRNAs may act as molecular markers of diseases to support the diagnosis. Additionally, circRNAs are strongly associated with diseases and clinicopathological features, which may enhance diagnostic and prognostic accuracy. In addition to clinical uses as biomarkers, circRNAs can also be developed as promising therapeutic targets.

However, there is still a long way to go before circRNAs can be used as disease biomarkers and therapeutic targets. First, the precise mechanisms of circRNAs are still ambiguous. For example, the mechanism involved in circRNA degradation and enrichment during exosome formation still needs to be studied. Second, even though a lot of disease-related circRNAs have been identified by bioinformatics models and databases, only a short list of circRNA candidates has been verified through rigorous functional and mechanistic experiments *in vitro* and *in vivo*. The clinical feasibility of circRNAs needs to be validated across multiple large cohort studies. Creating efficient circRNA-based therapeutic methods will also contribute to understanding clinical potential of circRNAs. Eventually, clinical application of circRNAs as disease biomarkers and therapeutic targets requires further investigations.
